# Rapid Multimuscle Cortical Mapping of the Upper Limb: Activity Reveals Intralimb Gradients

**DOI:** 10.1111/ejn.70579

**Published:** 2026-06-16

**Authors:** Rowan Boyles, Napat Kiatwongwanich, Mikal Vicente, Paul H. Strutton

**Affiliations:** ^1^ The Nick Davey Laboratory, Department of Surgery and Cancer Imperial College London London UK; ^2^ Imperial College Healthcare NHS Trust London UK; ^3^ Centre for Vestibular Neurology, Department of Brain Sciences Imperial College London London UK

**Keywords:** corticospinal excitability, motor cortex mapping, motor evoked potentials, neuronavigation, transcranial magnetic stimulation, upper limb

## Abstract

Transcranial magnetic stimulation (TMS) mapping provides valuable insight into corticospinal organisation and plasticity, but conventional protocols are often too time‐consuming to be practical in clinical settings. This study aimed to refine a rapid, multimuscle TMS mapping protocol capable of comprehensively characterising upper limb motor representations, including both distal and proximal muscles, and to examine how stimulation intensity and voluntary muscle activity shape cortical map metrics and motor evoked potential (MEP) latency. Nineteen healthy adults underwent neuronavigated TMS mapping of eight upper limb muscles using a rapid random walk protocol. Cortical maps were acquired at multiple stimulation intensities referenced to the motor threshold of the first dorsal interosseous muscle, both at rest and during a task requiring low levels of muscle activity. Map area, volume and centre of gravity were quantified using relative and absolute thresholds, alongside automated analysis of MEP onset latency. Cortical representations showed stable somatotopic organisation across conditions, with proximal‐muscle centres of gravity located more medially than distal muscles. Increasing stimulation intensity preferentially expanded distal muscle map area and volume, whereas voluntary muscle activity selectively facilitated proximal‐muscle representations, even after controlling for background muscle activity. These effects revealed a clear proximal–distal gradient in the physiological modulation of cortical maps. MEP latencies were shorter for proximal muscles and during active contraction. At rest, latency increased with distance from the map centre for several muscles, but this spatial gradient was abolished or inverted during voluntary activity. These findings demonstrate that rapid, multimuscle cortical mapping is feasible and yields physiologically meaningful metrics across the upper limb. The results highlight a functional dissociation between proximal and distal representations and underscore the importance of combining high‐intensity resting and active mapping conditions to obtain a complete assessment of corticospinal integrity, with direct relevance for future clinical studies in neurological populations.

## Graphical abstract

Rapid cortical mapping of multiple muscles is feasible with potential for clinical applications. Random walk TMS cortical mapping was carried out for 8 upper limb muscles simultaneously at rest or with muscle contraction. Centres of gravity were somatopic. Distal muscle map areas were affected by stimulator intensity, proximal muscle map areas by activity.

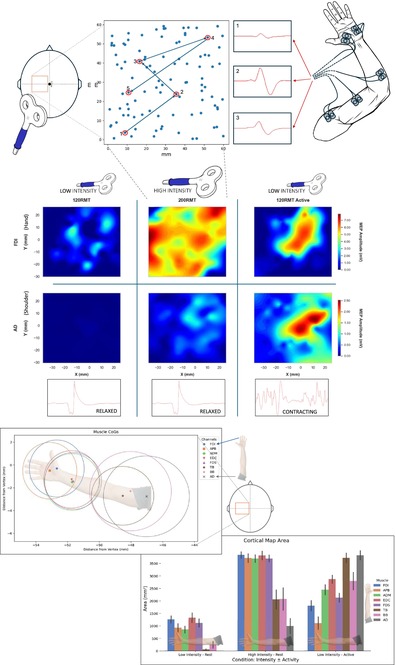

**Abbreviations**
ad
: anterior deltoid
ADM: abductor digiti minimi
APB: abductor pollicis brevis
BB: biceps brachii
BG: background contraction
CED: Cambridge Electronic Design
CoG: centre of gravity
CST: corticospinal tract
CV: coefficient of variation
EDC: extensor digitorum communis
EMG: electromyography
FDI: first dorsal interosseous
FDS: flexor digitorum superficialis
fMRI: functional magnetic resonance imaging
GRASSP: graded redefined assessment of strength, sensation and prehension
ICREC: Imperial College Research Ethics Committee
IQR: interquartile range
M1: primary motor cortex
MEP: motor evoked potential
MNI: Montreal Neurological Institute
MRI: magnetic resonance imaging
MSO: maximum stimulator output
MVC: maximal voluntary contraction
NIHR: National Institute for Health and Care Research
RMS: root mean square
RMT: resting motor threshold
SCI: spinal cord injury
TB: triceps brachii
TMS: transcranial magnetic stimulation
WLS: weighted least squares

## 1 Introduction

Transcranial magnetic stimulation (TMS) enables non‐invasive assessment of nervous system function through electromagnetic induction of action potentials in central or peripheral nerves (Siebner et al. 2022). TMS performed over the primary motor cortex elicits motor evoked potentials (MEPs), which can be used to infer the integrity of the corticospinal tract (CST) (Spampinato et al. 2023), the primary neural pathway underlying precise control of voluntary movement in humans (Levi and Schwab 2022). This facilitates detailed assessment of the changes in neurophysiology that occur after neurological injury and therefore has both diagnostic and prognostic implications (Davey et al. 1999; Davey et al. 1998; Arora et al. 2022). TMS delivered systematically over a larger area of the scalp enables construction of cortical maps, giving insight into the size and extent of muscle representations in the motor cortex (Freund et al. 2011; Cortes et al. 2017; Van De Ruit and Grey 2016). This method can be used to observe cortical plasticity in response to neurological injury (Arora et al. 2022; Freund et al. 2011).

The ability to examine the changes in upper limb neurophysiology is particularly important after neurological injury, such as acquired brain injury or spinal cord injury, since impairments in hand and arm function can significantly impact on independence and quality of life (Veerbeek et al. 2011; Anderson 2004). Previous studies have often focused on recording responses from single muscles, typically intrinsic muscles of the hand (Hupp et al. 2018; Balbinot et al. 2023; Stinear et al. 2017). However, upper limb function requires complex coordination between multiple muscle groups, controlling the action at several joints (Pregnolato et al. 2026; Tanzarella et al. 2024; Tresch and Jarc 2009; Saito et al. 2022). As such, a comprehensive neurophysiological assessment is warranted to effectively capture the extent of impairment at both gross and fine motor levels.

Despite their potential clinical value, mapping protocols involving multiple muscles can be time‐consuming, limiting their feasibility in clinical settings. One study carried out simultaneous mapping of four upper limb muscles in healthy people (Wassermann et al. 1992). This relied on a time‐intensive grid‐based mapping procedure with all maps recorded at 100% of maximum stimulator output (MSO). The potential widespread use of such a protocol is therefore limited by concerns for participant comfort, particularly in clinical populations. Stimulating at 100% MSO also limits the ability to examine muscle response profiles across different mapping conditions.

Recent advances in neuronavigation have led to the development of rapid mapping protocols that can be carried out in a few minutes (van de Ruit et al. 2015). These initially involved assessment of a single, distal muscle only (van de Ruit et al. 2015) but have more recently been applied to simultaneous mapping of multiple muscles (Yuasa et al. 2022). Cortical maps for seven upper limb muscles were produced simultaneously at a range of intensities, to determine the optimal stimulation intensity for mapping both proximal and distal muscles of the upper limb (Yuasa et al. 2022). Stimulation intensity was defined relative to the threshold for first dorsal interosseous (FDI) and all maps were recorded during relaxation, i.e., with no background muscle activity. Consequently, the authors were only able to obtain ‘optimal’ motor maps for the proximal muscles (triceps brachii [TB], biceps brachii [BB] and anterior deltoid [ad]) in just 13.3%–33.3% of their participants (Yuasa et al. 2022). Adjustments are therefore required to capture both distal and proximal upper limb muscle responses which is an aim of the present study.

Being able to obtain cortical maps from both distal and proximal muscles simultaneously would support better characterisation of motor cortex changes and corticospinal tract integrity after neurological injury. Before clinical studies can be undertaken, preliminary work is required in healthy participants to refine the protocol and to obtain reference data. Further to this, being able to reliably obtain responses from both proximal and distal muscles at the same time facilitates direct comparison of the motor cortex representations of these muscles under different conditions and is therefore important for our understanding of upper limb motor control.

The present study builds on previous multimapping studies by using stimulation intensities previously shown to obtain ‘optimal’ maps (Yuasa et al. 2022) to simultaneously map eight key muscles of the upper limb. The muscles chosen are in line with the detailed clinical assessment of upper limb function in the graded redefined assessment of strength, sensation and prehension (GRASSP); a comprehensive assessment of upper limb neurology and function in spinal cord injury (Velstra et al. 2014). As such, all key myotomes and planes of movement are represented and therefore are applicable to any clinical population with complex impairments affecting upper limb muscle synergies. Further to this, an active mapping condition was included, where participants maintained a constant low level of background muscle contraction. This facilitates MEPs and was intended to overcome the difficulty in obtaining responses from proximal upper limb muscles (Spampinato et al. 2023).

Since the present study aims also to provide normative reference data about upper limb cortical representations, previous results in the literature (Yuasa et al. 2022) are further extended by reporting intermuscle differences in map centre of gravity (CoG) and the topography of MEP latencies. MEP latency gives information about which neural populations underlie the measured response and helps to establish whether the same populations are being stimulated when the site of stimulation changes (Spampinato et al. 2023). We hypothesised that stimulation at the map periphery would engage less excitable intracortical circuits or slower, indirect descending pathways, resulting in increased latencies compared to the map centre. Because changes in latencies are also a marker of neurophysiological impairment (Davey et al. 1998; Arora et al. 2022), establishing this normative latency topography may serve as a relevant physiological marker after neurological injury. Furthermore, the combination of rapid mapping and automated latency detection (See Section 2) provides a uniquely powerful method to map these latency profiles at scale.

The study aims were as follows:

- Determine whether multiple upper limb muscles can be mapped rapidly and comprehensively, with meaningful MEP responses obtained from both distal and proximal muscles.
- Assess how cortical map metrics (area, volume and CoG) differ between upper limb muscles in healthy participants.
- Establish whether and how MEP latencies for a given muscle vary within its cortical map.
- Develop a rapid multimuscle mapping protocol for deployment in neurological populations.
- Provide normative reference data for comparison in future clinical studies.

## 2 Methods

### 2.1 Participants

Nineteen healthy adult volunteers were recruited from the authors' personal and professional networks (11 female, one left‐handed, mean age 24.68 years, SD 4.58, range 19–38). Safety screening was carried out prior to participation in accordance with established guidelines (Rossi et al. 2011; Rossi et al. 2021); no participants had contraindications to TMS. Participants gave their written informed consent prior to participation. Ethical approval for the study was provided by the Imperial College Research Ethics Committee (ICREC), study number 6386803.

### 2.2 Electromyographic (EMG) Recording

Eight pairs of adhesive skin surface Ag/AgCl electrodes (self‐adhesive, 2 cm diameter, Ambu A/S, Ballerup, Denmark) were attached to the skin overlying eight muscles of the dominant upper limb: first dorsal interosseous (FDI), abductor pollicis brevis (APB), abductor digiti minimi (adM), extensor digitorum communis (EDC), flexor digitorum superficialis (FDS), triceps brachii (TB), biceps brachii (BB) and anterior deltoid (AD). These were chosen to give good coverage of myotomes and planes of movement, as well as reflecting muscle groups assessed in neurological populations, such as those with spinal cord injury (Velstra et al. 2014). A single ground electrode was attached to the ulnar styloid process on the nondominant limb. Prior to attaching the electrodes, the skin surface was prepared by cleaning with an isopropyl alcohol swab.

EMG signals were amplified (×1000) and filtered (10–1000 Hz; Digitimer 360, Digitimer, Welwyn Garden City, UK) and sampled at 2 KHz by an analogue to digital converter (Power 1401, Cambridge Electronic Design [CED], Cambridge, UK) with real time monitoring via CED Signal on a Windows PC. Maximal voluntary contraction (MVC) was recorded for all eight muscles consecutively. For each muscle, the experimenter demonstrated the target movement and assisted the participant to stabilise the muscle origin. The participant was then instructed to contract maximally against the experimenter's resistance for 3–5 s after a countdown. The experimenter provided verbal encouragement during contraction to ensure full effort and the resulting EMG was captured in CED Signal as consecutive 0.75 s frames. This was repeated three times for each muscle. MVC was calculated by extracting the peak root mean square (RMS) values of the EMG for all frames and averaging the top 3 values. MVC recordings were typically completed at the end of the experiment after TMS mapping and took approximately 5 min. They were not completed for three participants due to time constraints.

### 2.3 Neuronavigated TMS

TMS was carried out in accordance with current guidance regarding safety and methodological quality (Rossi et al. 2021; Chipchase et al. 2012). Motor cortical stimulation was carried out using a Magstim 200^2^ and a figure‐of‐8 coil (loop diameter 70 mm, The Magstim Company, Whitland, UK), with real time visual feedback of coil position provided by a BrainSight neuronavigation system (BrainBox Ltd., Cardiff, UK). The participant's head was registered to the system using a standard MRI template, the MNI head model (Mazziotta et al. 2001), scaling to the nasion and bilateral preauricular points. Prior to stimulation, the participant's vertex was measured as the intersection of two lines, one from nasion to inion and one from tragus to tragus, marked and recorded in the BrainSight system. During stimulation, relative positions of the participant's head and the TMS coil were continually tracked, with each stimulation location recorded in MNI space.

FDI hotspot was determined by stimulating over the primary motor cortex (M1) of the participant's dominant hemisphere (18 left and 1 right), starting over the ‘hand knob’ (Wassermann et al. 1996) as identified on the MNI brain template. Hotspot was defined as the point over which TMS produced the largest peak‐to‐peak MEP amplitude. Resting motor threshold (RMT) for FDI was then determined using standard methods, stimulating over the hotspot at different intensities in blocks of 6, until peak‐to‐peak amplitude exceeded 0.05 mV for 3 out of 6 MEPs (Conforto et al. 2004; Rossini et al. 1994; Rossini et al. 2015).

**FIGURE 1:**
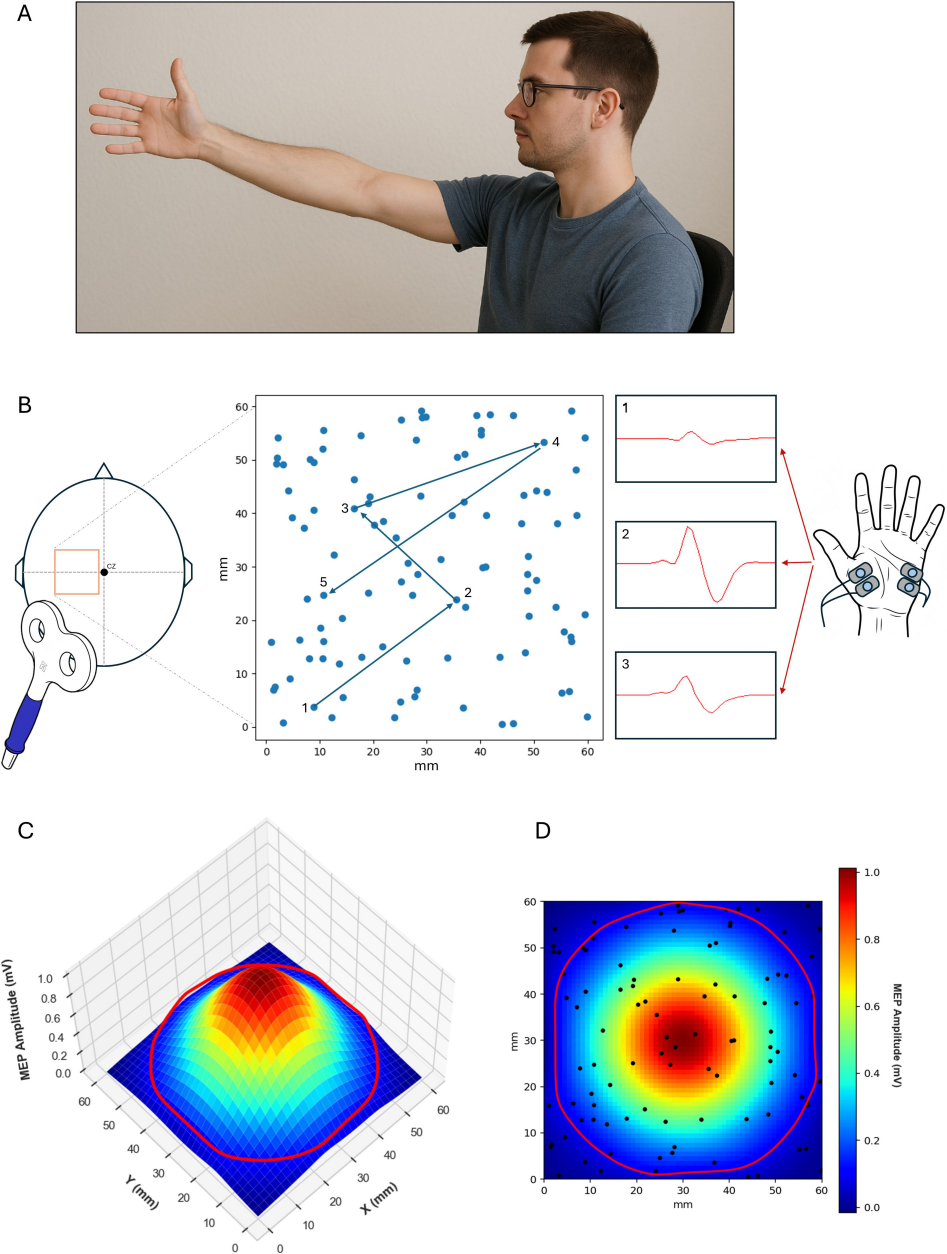
Visual summary of TMS mapping protocol (schematic only). (A) Example of upper limb task. (B) Random walk mapping technique (van de Ruit et al. 2015), simulated data. Map grid boundaries are defined within the neuronavigation software, and stimuli are delivered pseudorandomly within these limits. Arrows highlight the pseudorandom nature of stimulus delivery. Example MEPs depicted are from a single muscle—eight muscles are recorded simultaneously. (C) Simulated 3D heat map of MEP responses from a single muscle over mapped area. Red ring denotes threshold for area calculation. Volume calculated by summing MEP values in area above threshold. MEP amplitude mV (simulated data). (D) Top‐down view of C (Images of upper limb task, TMS coil and hand generated by Chat GPT, Open AI).

**FIGURE 2:**
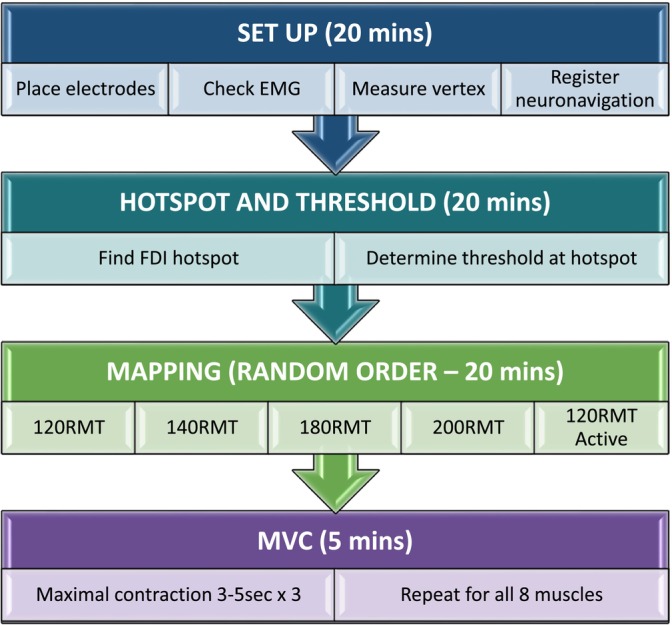
Flow chart of mapping process with estimated time for each stage. Hotspot and threshold involved delivering approximately 30–100 pulses. Eighty pulses were delivered for each map (400 total for five maps).

A 6 × 6 cm target grid was placed on the model brain within BrainSight centred over the identified FDI hotspot, with the edge parallel to the longitudinal fissure. This area was mapped using the random walk method, with 80 stimuli delivered at approximately 0.5 Hz, achieving even coverage (van de Ruit et al. 2015) (Figure 1B). This was performed four times at rest for each participant; at 120%, 140%, 180% and 200% of FDI RMT, plus one further active map at 120% of RMT with upper limb muscles contracted. These will be referred to as 120RMT, 140RMT and so on throughout the rest of this paper; each mapping condition will subsequently be referred to as a ‘session’. The intensities used were previously shown to be appropriate for obtaining optimal map areas from the different muscles of the upper limb (Yuasa et al. 2022). The order of these five mapping sessions was randomised for each participant. For the active mapping session, participants were asked to hold their arm out at 90° in front of them with elbow extended and fingers spread wide (Figure 1A). This task was chosen to enable cocontraction and simultaneous activation of all upper limb muscles. Since simultaneous biofeedback from eight muscles would be challenging for participants, they were simply instructed to maintain a constant contraction. Stimuli in the active session were then delivered in blocks of 10 with participants resting between blocks to limit the effect of fatigue. Stimuli in the resting sessions were delivered continuously unless the participant requested a break. Due to variations in motor threshold, it was not possible to complete all five sessions in all participants, see Table 1. A flow chart of the mapping process and time taken for each step is provided in Figure 2.

**TABLE 1:** Numbers of participants with completed maps at each stimulation intensity.

Percent of FDI RMT	Participants tested (%)
120^a^	19 (100%)
140	17 (89.5%)
180	17 (89.5%)
200	12 (63.2%)

^a^ Relaxed and active.

### 2.4 Analysis

#### 2.4.1 Map Creation

**FIGURE 3:**
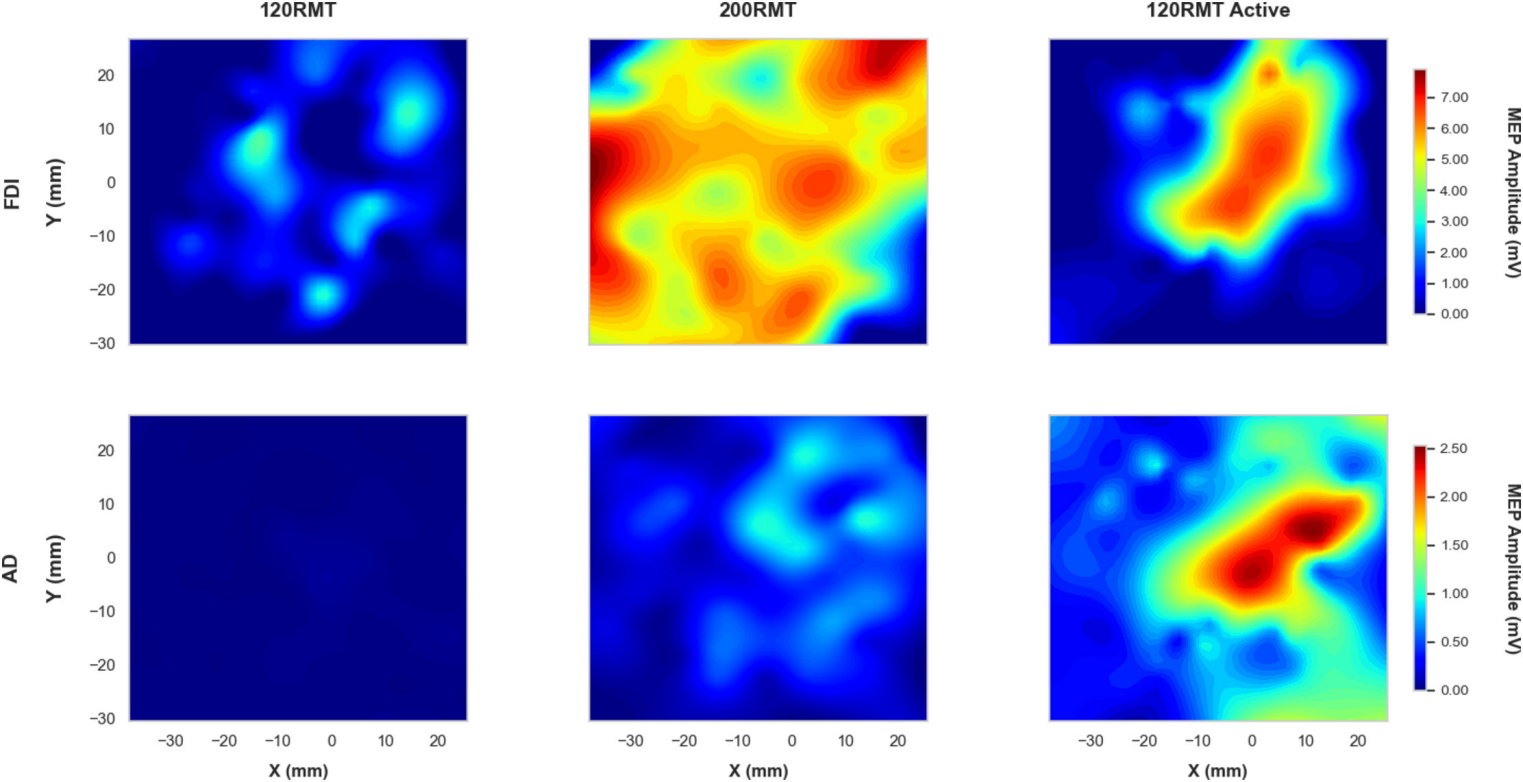
Example cortical maps from one participant. One proximal (AD) and one distal (FDI) muscle at low and high resting intensities and in the active condition. All plots are centred at FDI hotspot. Proximal muscles show greater facilitation with activity, distal muscles with increased intensity. Note the difference in amplitude scale between FDI and AD.

Cortical maps were analysed using the method described previously (van de Ruit et al. 2015; van de Ruit 2016), implemented in Python. For all stimulation locations, positional outliers were defined as those falling outside the 99% prediction interval with respect to coil orientation relative to the scalp. These were excluded from further analysis (3.7% of all samples). For EMG data, outliers were defined as any stimulation epochs where the MEP amplitude exceeded 3.5SD of the mean for that map, or anywhere where the RMS amplitude of prestimulus EMG (100 ms before the TMS pulse) was more than 2SD greater than the mean prestimulus RMS amplitude for that map. These epochs were also excluded (3.8% of all samples). A linear plane of best fit was fitted through the array of stimulation locations, with each stimulation point moved to the nearest point on the plane. The Gridfit function (D'Errico 2024) was used to create a surface contour plot of MEP amplitude over this plane. To reduce extrapolation artefacts at the map periphery, fitted values outside the convex hull of sampled stimulation points were attenuated before map metrics were calculated, whereas values within the sampled hull were left unchanged. The resulting 3D surface was used to determine area, volume and CoG for each muscle at each stimulation intensity (Figure 1C,D). Representative maps from one participant are shown in Figure 3.

#### 2.4.2 Area and Volume (Relative and Absolute Thresholds)

Previous mapping studies used a relative threshold, normalised to a muscle's maximum MEP amplitude, for map area calculations, to allow direct comparison across maps recorded at different intensities or activity levels (Van De Ruit and Grey 2016; van de Ruit et al. 2015). Since the present study compares simultaneous maps of different muscles, using a relative or normalised threshold risks obscuring absolute intermuscle differences in map extent. As such, area and volume were calculated using both relative and absolute thresholds. For the relative threshold, area was calculated as the sum of all grid segments where the MEP amplitude was greater than 10% of the maximum amplitude for that muscle across all mapping sessions. Amplitudes on the fitted grid were normalised using a linear ‘hinge’ transformation. The threshold point (10% of max amplitude) was defined as 0, and the maximum amplitude was assigned a value of 1. Grid points with amplitudes below threshold contributed 0, whereas suprathreshold amplitudes were linearly rescaled between 0 and 1. Written mathematically, if Ai = fitted MEP amplitude at grid location i, $A_{\max}$ = maximum MEP amplitude for that muscle and τ= threshold fraction (0.1, i.e., 10%), the normalised amplitude ($A_{n}$) is given by

$$A_{n}=\max\left(0,\min\left(1,\frac{\mathrm{Ai}-\tau A_{\max}}{\left(1-\tau \right)A_{\max}}\right)\right)$$

Relative volume was calculated by summing these normalised amplitudes over the area (nAmp · mm^2^). The absolute threshold for MEP presence was set at 0.05‐mV peak‐to‐peak amplitude (Rossini et al. 1994; Rossini et al. 2015). Absolute area was calculated as the sum of all grid segments, which exceeded this. Absolute volume (mV·mm^2^) was calculated by summing all the fitted MEP amplitudes over this area.

#### 2.4.3 CoG

CoG is defined as the weighted mean average of the MEP amplitude values (*z*) in the *x* and *y* dimensions of the grid,

$${\mathrm{CoG}}_{x}=\frac{\sum_{i}x_{i}z_{i}}{\sum_{i}z_{i}},\;{\mathrm{CoG}}_{y}=\frac{\sum_{i}y_{i}z_{i}}{\sum_{i}z_{i}}$$

${\mathrm{CoG}}_{x}$ was inverted for the left‐handed participant. To analyse spatial organisation of CoGs, Euclidean distance (D) from the vertex was calculated for each muscle CoG by combining the lateral (*x*) and anterior–posterior (*y*) distances.

$$D=\surd \left(x^{2}+y^{2}\right)$$

#### 2.4.4 MEP Latency

MEP onset latencies were calculated using a Python script based on a previously published algorithm (Bigoni et al. 2022). Physiologically implausible values were excluded using a pooled standard deviation (SD) filter and known MEP latency ranges from the literature (Bastani Jahromi and Jaberzadeh 2012; Sollmann et al. 2017; Yook et al. 2011; Inoue et al. 2003). To avoid outlier masking in participants with small trial counts, a ± 2.5 SD threshold was derived from the cohort‐level pooled SD for each muscle and condition and calculated relative to each participant's mean latency (1.57% of trials removed). After SD filtering, retained latency values that lay outside the plausible range of known MEP latency values for that muscle were also excluded (5.7% of trials). To examine the relationship between MEP latency and stimulation location, the Euclidean distance from every stimulation point to each muscle CoG was determined. To prevent bias from sparsely sampled sites far from the CoG, stimulus locations more than 50 mm away were excluded. Only latencies from maps recorded at 180RMT and 200RMT were included for the analysis of resting conditions, since proximal‐muscle responses were typically absent at lower intensities and latency estimates would therefore have been biased in these maps. Despite this, the dataset for latency analysis comprised over 19,000 trials.

### 2.5 Statistics

Where MVC measurements were available, background contraction level was expressed as a proportion of MVC (BG). Resting BG across 120RMT, 140RMT, 180RMT and 200RMT was summarised descriptively using participant‐level means and within‐participant SDs across intensities. Because resting BG was low and stable (see Section 3), these intensities were collapsed into a single rest level for inferential analysis. BG differences between rest and active conditions were analysed using a linear mixed‐effects model with fixed effects of Muscle, Task (rest vs. active), and their interaction, and a random intercept for Participant.

For CoG analyses, Euclidean distance from the vertex was analysed using a linear mixed‐effects model with fixed effects of Muscle and Session and a random intercept for Participant. Because proximal‐muscle CoGs from 120RMT and 140RMT were excluded owing to inconsistent MEP elicitation, the resulting Muscle × Session structure contained empty cells and CoG distance was analysed using an additive rather than interaction model. Significant effects were followed by Holm‐adjusted model‐based estimated marginal mean contrasts.

Cortical map area and volume were analysed separately for relative‐ and absolute‐threshold metrics. Because repeated observations were nested within participant, the primary inferential framework for all map‐metric analyses was a linear mixed‐effects model with Participant modelled as a random intercept. For resting‐condition analyses, fixed effects of Muscle and Intensity were fitted, and monotonic intensity trends were examined in separate models treating intensity as a numeric predictor to estimate muscle‐specific slopes. For rest‐versus‐active analyses, models were fitted both without adjustment and with centred BG as a continuous covariate. For change analyses, delta values were defined as the metric at each session minus the corresponding 120RMT value within each Participant × Muscle pair; these were analysed using mixed‐effects models with fixed effects of Muscle and Session, with centred ΔBG included where available.

Because residual diagnostics indicated heteroscedasticity in several area and volume analyses, all map‐metric models were accompanied by weighted least squares (WLS) sensitivity analyses using Participant fixed effects and inverse‐variance weights derived from per‐cell residual variances in the corresponding diagnostic model. Primary mixed‐model and WLS results were compared to assess robustness to variance structure. Where the two approaches differed materially, findings were interpreted as sensitive to variance structure and treated cautiously rather than replacing the primary mixed‐effects result with the sensitivity analysis. Omnibus effects were tested using grouped Wald tests, and pairwise estimated marginal mean contrasts and simple‐slope estimates were Holm‐adjusted.

MEP onset latency was analysed in a separate trial‐level mixed‐effects pipeline. Trials with stimulation distances greater than 50 mm from the muscle CoG were excluded (< 0.001% of all trials), and analyses were restricted to 180RMT and 200RMT resting maps and the 120RMT active map. An activity‐versus‐rest mixed model and a progressive series of latency–distance mixed models were then fitted, with Participant included as a random intercept. The final saturated model was used to derive muscle‐ and condition‐specific simple effects.

## 3 Results

### 3.1 FDI Threshold

Mean FDI threshold across participants was 46.32% of MSO, SD 10.88, range 28%–78%. Since maps were recorded at various multiples of FDI threshold, multiples exceeding 100% MSO could not be completed and were therefore omitted for some participants (Table 1).

### 3.2 Background Activity in Resting and Active Conditions

**FIGURE 4:**
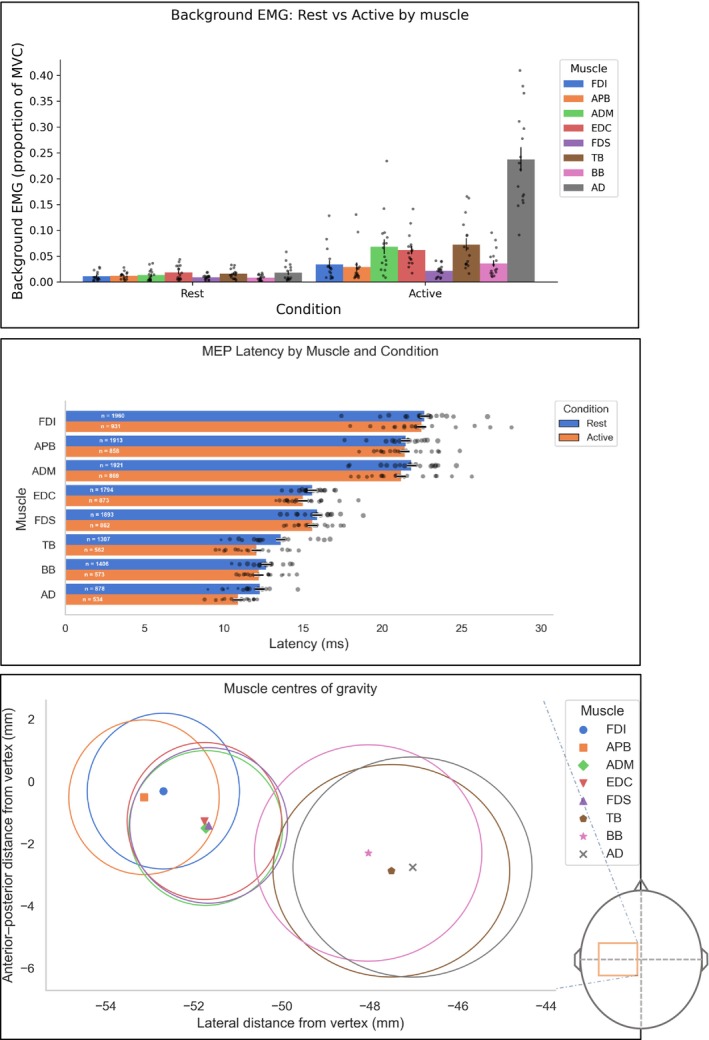
Top: Level of background contraction as a proportion of MVC in resting (all intensities averaged) and active mapping conditions. Middle: Mean MEP onset latency by muscle and session (rest or active). Bars show estimated marginal mean MEP latency for each muscle and condition from the trial‐level mixed‐effects model, with error bars indicating standard errors. Jittered points show participant‐level mean latencies for each Muscle × Condition combination. Point size is scaled according to the number of latency observations contributing to each participant mean—larger points indicate combinations with more contributing trials. Bar labels indicate the total number of latency observations contributing to each Muscle × Condition estimate. Bottom: Mean centres of gravity for different upper limb muscles, relative to vertex. Ellipses indicate 95% confidence intervals of each mean. CoGs from proximal muscles (TB, BB and AD) from low intensity conditions (120RMT and 140RMT) were not included as MEPs were not consistently elicited at these intensities.

Mean background EMG activity across all muscles was low at 1.3% of MVC at rest. Variability across resting sessions was also low; overall median within‐subject SD across resting intensities was 0.13% MVC (IQR 0.051% to 0.332%; *n* = 120). This supported collapsing resting sessions for the analysis. Background EMG increased with activity (mean 7.0% MVC, *p* = 0.045) and there was a Muscle × Task interaction (*p* = 2.12x10^−50^). AD showed a markedly larger active BG than all other muscles (23.7% MVC, *p* = 1.69x10^−34^). TB (7.2% MVC, *p* = 0.036) and adM (6.8% MVC, *p* = 0.043) had a slightly larger active BG than the remaining muscles, which ranged from 2.9% to 6.2% of MVC. However, in APB (2.9% MVC, *p* = 0.138) and FDS (2.1% MVC, *p* = 0.280) active BG was not significantly different from rest (Figure 4, top). Post hoc assessment of prestimulus RMS during the active maps showed moderate within‐map variability (median CV 34.4%, IQR 25.7% to 43.3%). BG was included as a covariate in the analyses of cortical map area and volume, ensuring that differences in mapping measures observed in the active condition were not due solely to differences in BG.

Further supporting the validity of the active condition, MEP latencies were shorter during muscle activity than at rest (main effect of condition: Δ = − 0.63 ms, *p* < 0.001). This effect varied by muscle (Condition × Muscle interaction: *p*< 0.001)—post hoc comparisons confirmed significant active‐rest shortening in all muscles except APB after Holm correction. FDS did exhibit shortening in the active condition even though it did not show a significant increase in background EMG (Figure 4, middle).

### 3.3 Muscle CoG

CoGs showed a somatotopic organisation with proximal‐muscle CoGs located more medially than distal muscle CoGs (Figure 4). Statistical analysis revealed a strong main effect of muscle (Wald *χ* ^2^(7) = 250.09, *p* = 2.65 × 10^−50^) but no main effect of session type (Wald *χ* ^2^(4) = 7.25, *p* = 0.123), indicating muscle CoGs were distinct from one another and stable across stimulation conditions. Pairwise comparisons indicated that the effect of muscle was driven by large differences between proximal muscles (ad, BB and TB) and hand/forearm muscles (FDI, APB, ADM, EDC and FDS). Proximal muscles were clustered together closer to the vertex (CoG positions were not statistically distinct from each other), whereas most hand/forearm muscles were clustered further away. The only hand muscle which did not cluster with any nonhand muscle was APB, which only clustered with FDI. As can be seen in the CoG plot (Figure 4), ADM grouped more closely with forearm muscles (EDC and FDS) than with the other intrinsic hand muscles (FDI and APB).

### 3.4 Map Area

#### 3.4.1 Relative Threshold

##### 3.4.1.1 Resting Maps

There were significant main effects of Muscle (Wald *χ* ^2^(7) = 108.09, *p* = 2.28 × 10^−20^) and Intensity (Wald *χ* ^2^(3) = 177.29, *p* = 3.40 × 10^−38^) and a significant Muscle × Intensity interaction (Wald *χ* ^2^(21) = 91.80, *p* = 7.89 × 10^−11^). This demonstrates that muscle cortical map areas differ between muscles and increase in size with increasing stimulation intensity but do not expand uniformly as stimulation intensity increases. Analysis of linear trends revealed a proximal‐to‐distal gradient in cortical excitability: The rate of map expansion per 20% increase from RMT ranged from 243 mm^2^ for ad to 739 mm^2^ for ADM (Table 2).

**TABLE 2:** Changes in area for all muscles by intensity and activity. Definitions of relative and absolute threshold for area calculation are provided in Methods—Analysis above.

	Relative threshold		Absolute threshold	
Muscle	Change in area per 20% RMT (mm^2^(SE))	Active—rest area difference, BG‐adjusted (mm^2^(SE))	Change in area per 20% RMT (mm^2^(SE))	Active—rest area difference, BG‐adjusted (mm^2^(SE))
FDI	648.5 (50.8)*	493.9 (234.8)*	415.9 (50.5)*	707 (203.60)*
APB	710.9 (50.8)*	192.0 (234.0)	493.2 (50.5)*	1012.2 (202.86)*
ADM	738.5 (50.8)*	1458.5 (245.0)*	522.1 (50.5)*	1281.0 (212.55)*
EDC	622.2 (50.8)*	1331.5 (239.8)*	509.0 (50.5)*	1407.6 (207.97)*
FDS	669.6 (50.8)*	975.8 (233.3)*	609.0 (50.5)*	1064.7 (202.23)*
TB	525.0 (50.8)*	3477.9 (244.1)*	442.3 (50.5)*	2734.3 (211.71)*
BB	475.6 (50.8)*	2490.3 (235.5)*	533.4 (50.5)*	2793.7 (204.20)*
AD	243.1 (50.8)*	3099.4 (370.8)*	450.9 (50.5)*	3090.0 (323.58)*

*Note*: For the mixed‐model monotonic trend analyses, muscle‐specific slopes were derived from a common intensity term plus muscle‐specific interaction terms; accordingly, the simple‐slope SEs are identical across muscles.

* significant at *p* < 0.05.

##### 3.4.1.2 Active‐Rest Comparison

Sixteen participants with complete MVC data contributed to this analysis, and to all subsequent analyses with background contraction as a covariate. The effect of muscle activity on map area was investigated through a comparison of cortical map areas at *resting* 120RMT versus *active* 120RMT for each muscle. There were significant main effects of Muscle (Wald *χ* ^2^(7) = 84.01, *p* = 2.09 × 10^−15^) and Task (Wald *χ* ^2^(1) = 4.42, *p* = 0.035) and a significant Muscle × Task interaction (Wald *χ* ^2^(7) = 155.27, *p* = 3.17 × 10^−30^). The change from rest to active was significant for all muscles except APB. Change in area from rest to active was greater in proximal than in distal muscles (Table 2).

##### 3.4.1.3 Change Versus Rest 120RMT

**FIGURE 5:**
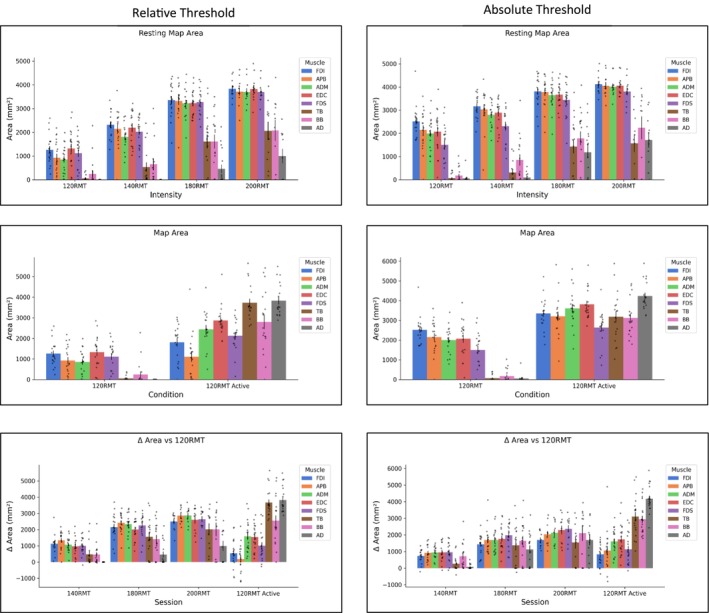
Bar charts of cortical map area. Left column shows areas calculated with relative thresholds (10% of max MEP amplitude for each muscle across all maps—rest and active). Right column show calculated using absolute threshold (0.05‐mV MEP amplitude). Results are clustered by stimulation condition (% of FDI RMT+/‐ activity) with muscle indicated by bar colour. Error bars denote SE. Top row shows resting maps only. Middle row shows 120RMT rest contrasted with 120RMT active. Bottom row shows difference in cortical map area between each condition and 120RMT (rest).

The relative effect of introducing activity was further examined by comparing the change in area between each mapping condition and 120RMT (Δ area; see Figure 5). The effect of muscle and condition on this change in area was then evaluated. Within‐session and within‐muscle comparisons were derived from the mixed‐effects model.

For the within‐session muscle comparisons; in the active condition, proximal muscles showed consistently larger Δ area than distal muscles: all nine proximal–distal pair comparisons were significant, with a mean proximal–minus–distal difference of +2163 mm^2^. By contrast, at 200RMT the pattern reversed, with larger Δ area in distal muscles over proximal muscles; three of nine proximal–distal pairs were significant (ad vs. all hand muscles), and the mean proximal–minus–distal difference was −1653 mm^2^.

For the within‐muscle session comparisons; in proximal muscles, Δ area was larger in active than 200RMT (ad and TB were significant), whereas for distal (FDI, APB, and adM) and forearm (FDS and EDC) muscles, Δ area was larger in 200RMT than in active with large, significant differences. Proximal‐muscle map areas increased more with activity, whereas distal/forearm map areas increased more with increasing intensity.

#### 3.4.2 Absolute Threshold

##### 3.4.2.1 Resting Maps

There were main effects of Muscle (Wald *χ* ^2^(7) = 404.51, *p* = 2.58 × 10^−83^) and Intensity (Wald *χ* ^2^(3) = 73.82, *p* = 6.47 × 10^−16^) but no Muscle × Intensity interaction (Wald *χ* ^2^(21) = 26.62, *p* = 0.184). In contrast to relative area, the linear trend of change in map area with change in intensity showed similar values across muscles, with no clear proximal–distal gradient (Table 2). However, the WLS sensitivity analysis showed a significant interaction effect (*p* = 0.005); this finding is therefore interpreted as nonrobust.

##### 3.4.2.2 Active‐Rest Comparison

There were significant main effects of Muscle (Wald *χ* ^2^(7) = 409.11, *p* = 2.65 × 10^−84^) and Task (Wald *χ* ^2^(1) = 12.06, *p* = 5.16x10^−4^) and a significant Muscle × Task interaction (Wald *χ* ^2^(7) = 123.32, *p* = 1.56 × 10^−23^). Every muscle showed a significant increase in area from rest to active, and this was more pronounced for proximal than distal muscles (Table 2).

##### 3.4.2.3 Change Versus Rest 120RMT

In the active condition, proximal muscles showed larger map area increases than distal muscles. Pairwise muscle comparisons showed a mean proximal–minus–distal difference of +1731 mm^2^ (mean across nine significant pairs). At 200RMT, there were larger map area increases in distal over proximal (mean proximal–minus–distal was −215 mm^2^), although pairwise differences were not significant after correction for multiple comparisons. Within‐muscle session comparisons showed active—200RMT was positive for proximal muscles (ad/BB/TB) but did not reach significance in ad or BB after correction, whereas distal and forearm muscles showed significant decreases (Active < 200RMT).

### 3.5 Map Volume

#### 3.5.1 Relative Threshold, Normalised Amplitudes

##### 3.5.1.1 Resting Maps

**FIGURE 6:**
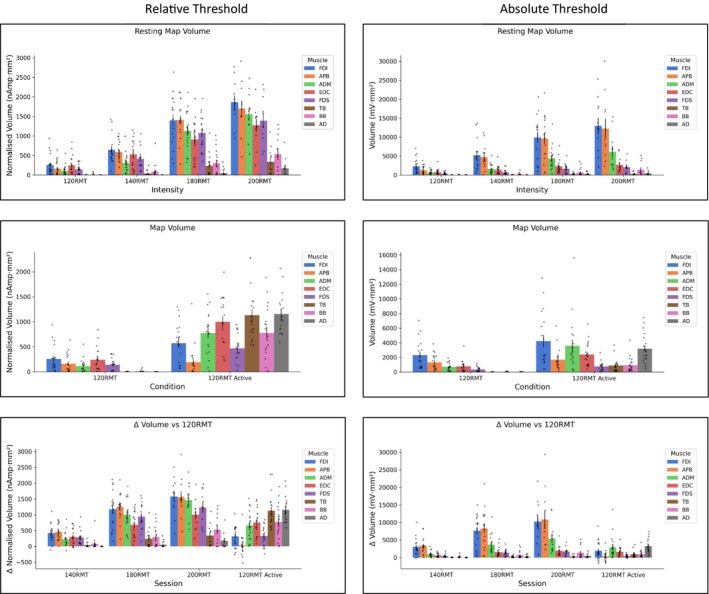
Bar charts of cortical map volume. Left column shows volumes calculated with relative thresholds (10% of max MEP amplitude for each muscle across all maps—rest and active, normalised amplitudes). Right column shows volumes calculated using absolute threshold (0.05‐mV MEP amplitude, raw MEP amplitudes). Results are clustered by stimulation condition (% of FDI RMT+/− activity) with muscle indicated by bar colour. Error bars denote SE. Top row shows resting maps only. Middle row shows 120RMT rest contrasted with 120RMT active. Bottom row shows difference in cortical map volume between each condition and 120RMT (rest).

There were significant main effects of Muscle (Wald *χ* ^2^(7) = 16.13, *p* = 0.024) and Intensity (Wald *χ* ^2^(3) = 254.58, *p* = 6.67 × 10^−55^) and a significant Muscle × Intensity interaction (Wald *χ* ^2^(21) = 249.38, *p* = 5.46 × 10^−41^) with proximal–distal variation in volume increases (Figure 6). Slopes were progressively steeper in the order ad, TB, BB, EDC, FDS, adM, FDI, and APB (Table 3).

**TABLE 3:** Changes in volume across muscle by intensity and activity. Definitions of relative and absolute thresholds for volume calculation are provided in Methods—Analysis above.

	Relative threshold		Absolute threshold	
Muscle	Change in volume per 20% RMT (nAmp · mm^2^ (SE))	Active—rest volume difference, BG‐adjusted (nAmp · mm^2^ (SE))	Change in volume per 20% RMT (mm^2^·mV (SE))	Active—rest volume difference, BG‐adjusted (mm^2^·mV (SE))
FDI	395.0 (24.9)*	280.3 (99.7)*	2569.6 (188.8)*	1293.2 (465.4)*
APB	395.1 (24.9)*	49.7 (99.3)	2672.9 (188.8)*	48.7 (463.8)
adM	364.3 (24.9)*	634.4 (103.9)*	1320.9 (188.8)*	1737.1 (485.7)*
EDC	241.2 (24.9)*	727.0 (101.8)*	462.6 (188.8)	769.4 (475.4)
FDS	315.8 (24.9)*	301.2 (99.0)*	425.3 (188.8)	151.4 (462.3)
TB	87.0 (24.9)*	1039.9 (103.5)*	52.4 (188.8)	−276.0 (483.8)
BB	122.6 (24.9)*	749.3 (100.0)*	291.5 (188.8)	395.1 (466.8)
AD	35.6 (24.9)	870.1 (156.8)*	83.1 (188.8)	−1034.8 (737.8)

*Note:* For the mixed‐model monotonic trend analyses, muscle‐specific slopes were derived from a common intensity term plus muscle‐specific interaction terms; accordingly, the simple‐slope SEs are identical across muscles.

* Significant at *p* < 0.05.

##### 3.5.1.2 Active‐Rest Comparison

There were significant main effects of Muscle (Wald *χ* ^2^(7) = 17.67, *p* = 0.014) and Task (Wald *χ* ^2^(1) = 7.91, *p* = 0.005) and a significant Muscle × Task interaction (Wald *χ* ^2^(7) = 70.89, *p* = 9.78 × 10^−13^). Map volume increases in the active condition were significant for all muscles except APB and larger in proximal than distal muscles (Table 3).

##### 3.5.1.3 Change Versus Rest 120RMT

Distal muscle map volumes were larger than proximal‐muscle map volumes at rest, and the difference increased with higher intensity: The mean proximal–minus–distal difference among significant pairs was −906 nAmp · mm^2^ at 180RMT (9/9 significant) and −1103 nAmp · mm^2^ at 200RMT (9/9 significant). In the active condition the effect reversed: The mean proximal–minus–distal difference was +652 nAmp · mm^2^, with 6/9 proximal–distal pairs significant.

The within‐muscle session comparisons show a similar pattern. Active—200RMT was negative for the distal group (mean = −1199 nAmp · mm^2^, 3/3 distal muscles significant) and forearm (−964 nAmp · mm^2^ in FDS; EDC not significant). In contrast, proximal muscles showed positive active–200RMT differences, reaching significance in TB (+587 nAmp · mm^2^), with BB and ad also positive but nonsignificant.

#### 3.5.2 Absolute Threshold, Raw Amplitudes

##### 3.5.2.1 Resting Maps

There were significant main effects of Muscle (Wald *χ* ^2^(7) = 16.46, *p* = 0.021) and Intensity (Wald *χ* ^2^(3) = 186.04, *p* = 4.38 × 10^−40^) and a significant Muscle × Intensity interaction (Wald *χ* ^2^(21) = 235.74, *p* = 2.95 × 10^−38^). Rate of change in volume per unit increase in intensity was high for distal muscles and negligible for AD and TB (Table 3). WLS sensitivity analysis supported the same overall interaction but suggested somewhat stronger evidence for smaller positive slopes in EDC, FDS and BB, indicating that these lesser slope effects were sensitive to variance structure.

##### 3.5.2.2 Active‐Rest Comparison

In the BG‐adjusted primary mixed‐effects model, absolute‐threshold volume showed significant main effects of Muscle (Wald *χ* ^2^(7) = 44.94, *p* = 1.41 × 10^−07^) and Task (Wald *χ* ^2^(1) = 7.72, *p* = 0.005) and a significant Muscle × Task interaction (Wald *χ* ^2^(7) = 21.17, *p* = 0.003). However, within‐muscle contrasts indicated significant active‐rest increases only in FDI and adM. In the WLS sensitivity analysis, ADM remained significant, whereas EDC, rather than FDI, reached significance. Activity‐related increases in absolute‐threshold volume are therefore limited and muscle‐specific, with some sensitivity to variance structure.

##### 3.5.2.3 Change Versus Rest 120RMT

In the session‐wise channel comparisons, distal muscles map volumes were consistently larger than proximal‐muscle map volumes at 140RMT, 180RMT and 200RMT; the mean proximal–minus–distal difference was approximately −2143, −5729 and −6983 (mV·mm^2^), respectively, with all proximal–distal pairs significant at 180RMT and 200RMT. In the active condition this proximal–distal difference persisted but was smaller (−1331 mV·mm^2^, with only two proximal–distal pairs significant). The within‐muscle session comparisons show a similar trend: 200RMT was greater than active and significant for several distal (and some forearm) muscles, whereas no proximal muscle showed a significant increase in active versus 200RMT.

### 3.6 MEP Latency

To investigate determinants of MEP latency, trial‐level mixed‐effects linear models were fitted to 19,134 observations. The estimated mean resting latency for FDI was 22.65 ms; latencies were significantly shorter for all other muscles (*p* < 0.001). Proximal muscles exhibited the most pronounced differences; Deltoid (ad) and Biceps (BB) resting latencies were 12.25 and 12.66 ms, respectively (Figure 4, middle). MEP latencies were reduced by an average of 0.63 ms (*p* < 0.001) during the active state compared to rest (Figure 4, middle).

**FIGURE 7:**
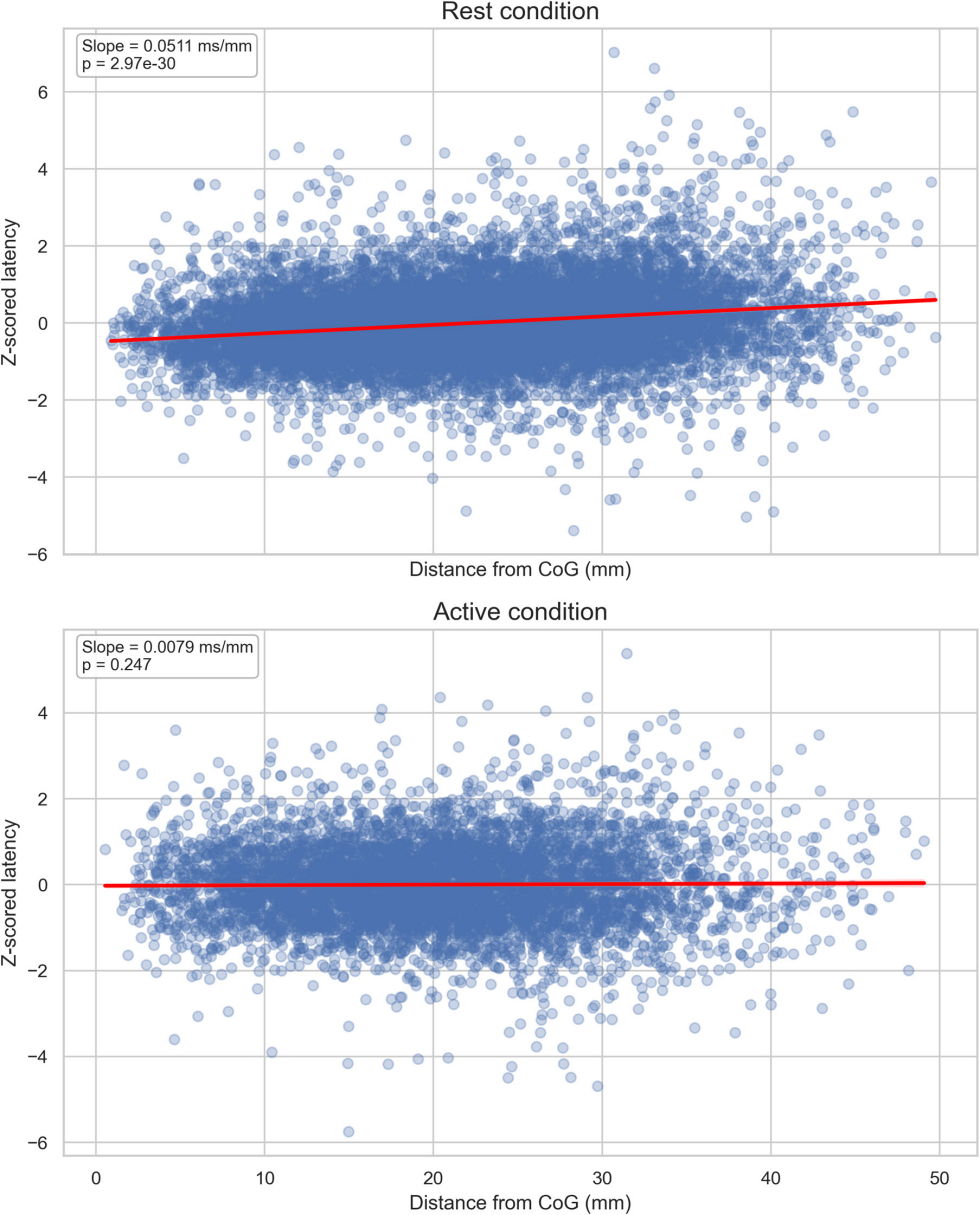
The relationship between MEP latency and stimulation distance. Scatter plots show *Z*‐scored latency versus distance from the muscle's CoG for all trials in the rest (top) and active (bottom) conditions. The red line represents the linear mixed‐model fit.

In the pooled distance‐by‐condition model the relationship between distance from CoG and latency was modified by the state of activity. In the rest condition, a significant positive relationship was observed (0.051 ms/mm, *p* < 0.001); latencies increased with increasing distance from the CoG. However, the distance by condition interaction was significantly negative (−0.043 ms/mm, *p* < 0.001) indicating that the distance‐dependent delay was effectively abolished during contraction; latencies were more uniform across the map (Figure 7).

**FIGURE 8:**
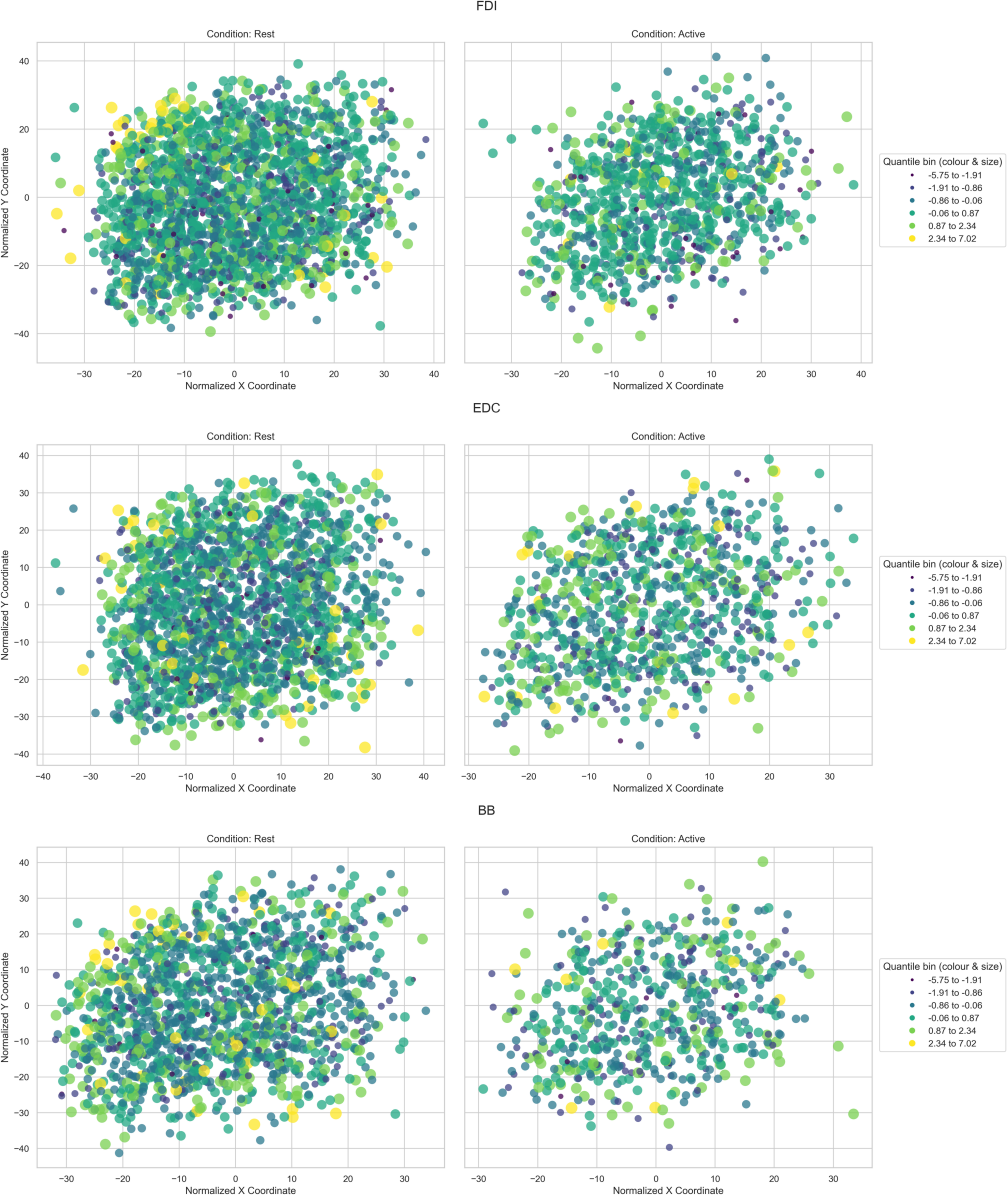
Illustrative topographic plots of *z*‐scored latency for a subset of muscles in rest and active conditions. Top figure shows FDI, middle figure EDC, bottom figure BB. Coordinates are normalised to the muscle CoG and latencies are expressed as Z‐score quantile bins.

The final saturated model revealed that the distance–latency relationship is muscle‐dependent. At rest, a significant positive relationship between distance and latency was observed in all muscles except FDI and ADM. The steepest distance‐dependent delays were found in BB and TB (Table 4). During background contraction, the significant delays observed at rest were abolished or diminished for all muscles. A significant inversion was observed for APB, indicating shorter latencies at the map periphery. BB and EDC retained slightly positive, significant slopes (Table 4). To illustrate, Figure 8 shows the spread of latencies across the map for FDI, EDC and BB.

**TABLE 4:** Latency–distance slope by muscle and condition. Positive numbers indicate longer latencies with increasing distance from the CoG; negative numbers indicate shorter latencies further from the CoG.

Muscle	Rest slope (ms/mm)	Rest *p*	Active slope (ms/mm)	Active *p*
FDI	+0.0017	0.724	−0.0048	0.495
APB	+0.0205	< 0.0001*	−0.0177	0.0200*
ADM	+0.0084	0.077	−0.0077	0.286
EDC	+0.0250	< 0.0001*	+0.0167	0.0233*
FDS	+0.0234	< 0.0001*	+0.0014	0.851
TB	+0.0835	< 0.0001*	+0.0054	0.549
BB	+0.0442	< 0.0001*	+0.0275	0.0042*
AD	+0.0314	< 0.0001*	+0.0182	0.0536

* The *p*‐values represent simple effects testing whether the absolute slope for each specific muscle‐condition combination differs from 0.

## 4 Discussion

This protocol‐development study showed that simultaneous mapping of eight upper limb muscles using a single hotspot and threshold reference is practical within a single 90‐min session and yields physiologically coherent outputs. Muscle CoGs were somatotopically arranged, in keeping with the classical model (Penfield and Boldrey 1937), and this was robust across different stimulation conditions (i.e., stimulator intensity and with or without muscle activity). The Traditional 1‐dimensional ‘homunculus’ view of M1 somatotopy has been challenged by animal studies using electrical microstimulation, where selective stimulation of different motor regions over varying time scales elicited specific motor patterns rather than isolated activation of effector muscles (Graziano 2016). Correspondingly, fMRI studies support an ‘integrate‐isolate’ model with somatotopic organisation of foot, hand and mouth representations into three concentric functional zones; distal parts of the effector (e.g., toes, fingers and tongue) are represented at the centre and more proximal parts (e.g., knee, shoulder and jaw) at the periphery. Effector regions alternate with ‘intereffector’ zones, which have an integrative role in movement planning and execution, including gross movement, postural control and physiological regulation (Gordon et al. 2023). Even so, Penfield's homunculus was intentionally schematic and described extensive overlap between regions but with an invariant proximal‐to‐distal order (cortically medial‐to‐lateral) (Penfield and Boldrey 1937). This order may reflect the resolution of different effectors competing for cortical space, with adjacency determined by both the physical body structure and movement repertoire (Graziano 2016). Although our data suggest that upper limb muscle cortical maps overlap, calculating CoGs necessarily simplifies these distributed data into a single metric, and these simplified metrics respect the proximal–distal order. Interestingly, adM occupied an intermediate position, clustering more closely with forearm than other hand muscles, potentially suggesting a stabilising role for this muscle. As such, these CoG data provide insight into the organisation of motor cortex in healthy individuals and could be useful in future studies examining changes in upper limb representations that occur in clinical populations, e.g., after SCI or stroke.

As demonstrated previously with maps recorded at MSO (Wassermann et al. 1992), proximal‐muscle map areas and volumes were smaller than distal muscle map areas at all stimulation intensities. Cortical map area and volume also increased with increasing stimulation intensity for all muscles, as previously observed for FDI (Van De Ruit and Grey 2016). However, the distinct profile of area change across different intensities differed between muscles: With a relative threshold for area calculation and normalised amplitudes, distal muscles showed larger increases in map area and volume per unit increase in intensity, relative to proximal muscles. This pattern changed when an active task was introduced. In line with studies showing increases in MEP amplitude (Hess et al. 1987) and steeper response curves (Kukke et al. 2014) for the target muscle when contracted, map area and volume also increased for all muscles during an active task. However, proximal‐muscle map areas and volumes increased more than distal, and this was true even when controlling for the level of background contraction. The task used here induced greater muscle activity in AD than any other muscle (23.7% of MVC), but the relative facilitation of map area was also observed in TB, which showed a much lower level of contraction across participants (7.0% of MVC).

When volume was calculated using a relative threshold, the proximal–distal split was clear, with larger increases in proximal‐muscle volumes during activity, even when controlling for level of background contraction, as observed for map area. In contrast, when volume was calculated using an absolute threshold and background contraction was included as a covariate, distal muscles showed much greater increases than proximal. This likely reflects the larger absolute MEP amplitudes in distal muscles relative to proximal muscles, which dominate absolute volume estimates. Although voluntary activity clearly facilitates proximal‐muscle MEPs on a normalised scale, these effects remain small in absolute units and are closely related to background contraction level. Consequently, adjusting for background contraction absorbs much of the variance associated with activity, removing the apparent effect of activity on absolute volume. This shows the importance of threshold selection and normalisation for proper comparison of intermuscle differences.

A previous study, which attempted to identify the optimal intensity parameters for measurement of cortical map area in different upper limb muscles, was not able to reliably obtain responses from proximal muscles, despite stimulating at high intensities (Yuasa et al. 2022). The fact that proximal‐muscle responses here were differentially facilitated by muscle activity, rather than stimulation intensity, suggests addition of an active task may be useful for probing cortical representations of proximal muscles. Regarding the reasons for this, facilitation of MEPs has previously been shown to be task dependent, with smaller MEPs recorded from rectus abdominis during a forced expiration task than a trunk flexion task, despite similar levels of EMG (Tunstill et al. 2001). Further, facilitation of erector spinae MEP was sensitive to cross‐facilitation effects in different motor tasks, with larger MEPs elicited during elbow flexion and index finger abduction than elbow extension and thumb abduction (Chiou et al. 2018). Corticospinal drive is therefore sensitive to motor context. In the present study, proximal muscles may have been selectively facilitated by a gross motor task that primarily involved placement of the limb rather than fine motor control. This is supported by the finding that during manual tasks MEP amplitude was facilitated more for the prime movers—distal muscles in grip and grasp tasks, proximal muscles in push and load tasks (Schieppati et al. 1996). This has applications in clinical research, since representations of hand and forearm muscles change after neurological injury (Freund et al. 2011; Tazoe and Perez 2021). Use of an active task in multimuscle rapid mapping may allow better characterisation of whole upper limb representation changes than maps measured only at rest. Accordingly, appropriate task selection is important, as failure to elicit facilitation in different prime movers may indicate specific failures within the motor system. Furthermore, motor imagery research suggests that in the absence of the ability to voluntarily activate muscles—a task involving thinking about movement could also facilitate MEPs to create useful maps (Grosprêtre et al. 2016). This is particularly relevant for people with profound weakness who may struggle to perform the activity in question.

The combination of rapid mapping and automated latency detection provides a powerful method of mapping latency profiles. Our data provide a robust window into the topography of MEP latency, with results based on over 19,000 MEPs. Proximal‐muscles' MEP latencies were shorter than distal, and latencies were shorter in the active condition, as shown previously (Furby et al. 1992). Analysis of the latency changes with distance from muscle CoG revealed a complex relationship that was dependent on both muscle and level of activity. In line with previous studies (Wassermann et al. 1992; Kallioniemi et al. 2015), there was an increase in latency with increasing distance from the CoG, but this was muscle specific, whereas latencies increased with distance from CoG for APB, EDC, FDS, TB and BB, and this was not observed for FDI or ADM. The effect of distance on latency at rest was generally subtle, with the difference from the centre to the edge of the map (≈3 cm) ranging from approximately 0.6 ms in APB to 1.3 ms in BB and 2.5 ms in TB. The muscles for which there was no effect may have a more even spread of direct corticospinal projections throughout the cortical representation. In the active state, the positive latency–distance gradient was generally diminished or abolished. There was a mild inversion of this relationship in APB with a trend towards shorter MEP latencies further from the CoG, whereas BB and EDC retained a small positive active‐state slope.

The observed disappearance of the positive latency–distance gradient during background contraction likely reflects a state of widespread physiological activation within the motor cortex and spinal motoneuron pool (Wilson et al. 1993). At rest, corticospinal output to a given muscle is expressed through a relatively quiescent system, such that stimulation of peripheral map locations may depend on less excitable intracortical circuits and on motoneuron pools further from firing threshold. Transmission in more distant sites may require greater temporal summation of descending volleys or engagement of later indirect pathways resulting in the significant delays observed in several muscles. By contrast, background contraction creates widespread facilitation across both motor cortex and the spinal motoneuron pool, reducing effective firing thresholds and diminishing spatial differences in excitability across the map. Under these conditions, a given corticospinal input can trigger motoneuron discharge more rapidly and consistently across stimulation sites, abolishing the distance‐dependent latency gradient observed at rest (Rossini et al. 1994). Regarding the inverted latency gradient during activity in APB, this muscle showed the lowest level of muscle activity and smaller relative facilitation of map metrics in the active condition. This may indicate that during background contraction, the central representation of APB was subject to surround inhibition intended to suppress unwanted movement during the task (Sohn and Hallett 2004; Beck and Hallett 2010; Beck and Hallett 2011). This inhibitory effect may be most concentrated at the map centre, effectively masking low‐threshold projections. Conversely, at the map periphery, where inhibitory control may diminish, faster conducting high‐threshold pathways remain available for recruitment, resulting in the observed negative slope where MEPs evoked at the edge of the map have shorter latencies than those evoked at the centre.

### 4.1 Limitations

Biofeedback was not included during the active condition which meant that it was difficult to control the level of muscle contraction. However, the aim was to activate all muscle groups simultaneously and biofeedback of all muscles may have been confusing for participants. This was a more straightforward task that could realistically be deployed in clinical settings with less specialist equipment. The task led to larger increases in ad muscle activity compared with other muscles, which may have influenced the relative effect of activity observed for proximal muscles. However, similar effects of activity were observed for TB and BB despite these showing similar levels of activation to more distal muscles. Intermuscle differences also remain despite including BG as a covariate, further suggesting that activity selectively enhances proximal‐muscle responses. Importantly, the addition of an active condition led to consistent proximal responses in a way that is not seen by simply increasing stimulation intensity.

Since the grid was centred on the FDI hotspot, the full extent of the cortical map for the other muscles may not have been fully explored. This represented a trade‐off between comprehensiveness of assessment, participant comfort and protocol time, as extending the grid further would have necessitated increasing the number of stimuli delivered. Previous studies identified 80 stimuli as the optimal number for pseudorandom cortical mapping over a 6 × 6 cm grid (van de Ruit et al. 2015). With five maps produced in total for most participants, this entailed at least 400 pulses—increasing this was not feasible. Despite this, recorded CoGs were approximately 1 cm apart for APB (most lateral) and ad (most medial) representations, suggesting that the extent of the mapping grid was sufficient to capture both proximal and distal muscle representations.

Although this study aimed to develop a protocol for clinical deployment and to obtain normative reference data from a healthy population, the mean age of participants was relatively young (24.68 years) and therefore may not be fully representative of clinical populations who would typically be older. However evidence suggests that age related changes in cortical maps are minimal at most (Hehl et al. 2020; Hehl et al. 2022; Patel and Chattha 2025) and any future studies in clinical populations would require control samples appropriately matched to the sample under study at that time.

### 4.2 Conclusion

This study demonstrates that rapid, multimuscle cortical mapping is feasible within a clinically realistic session and yields physiologically meaningful metrics of upper limb motor representations. The primary finding is a clear physiological dissociation in how these representations are modulated: Proximal‐muscle maps were preferentially facilitated by voluntary activity, whereas distal muscle maps were preferentially driven by stimulation intensity. A state‐dependent difference in MEP latency profiles was also observed, where the positive spatial gradient seen at rest was significantly attenuated or inverted during activity.

These findings have important implications. For motor neuroscience, they suggest a functional distinction in the cortical organisation of proximal and distal representations beyond simple somatotopy, reflecting their different roles in motor control. For clinical neurophysiology, our results strongly indicate that protocols aiming to assess the entire upper limb, particularly after neurological injuries like SCI or stroke, must incorporate both high‐intensity rest and lower intensity active conditions. Relying on only one state may provide an incomplete or misleading assessment of corticospinal integrity, especially for proximal muscles.

The next step is to apply this optimised protocol in clinical cohorts to validate its diagnostic and prognostic value. Even simple protocols assessing MEP presence or absence have shown clear prognostic utility in patients with stroke (Stinear et al. 2017). As such, a comprehensive mapping protocol may facilitate greater personalisation of care. Based on these findings, a simplified protocol using one high‐intensity resting map and one active map may be a promising approach for deployment in busy clinical settings. This work is currently underway.

## Author Contributions

**Rowan Boyles:** conceptualization, data curation, formal analysis, funding acquisition, investigation, methodology, software, visualization, writing – original draft, writing – review and editing. **Napat Kiatwongwanich:** data curation, investigation, software, visualization, writing – review and editing. **Mikal Vicente:** data curation, investigation, software, visualization, writing – review and editing. **Paul H. Strutton:** conceptualization, funding acquisition, investigation, methodology, resources, software, supervision, writing – review and editing.

## Funding

This study was made possible by grants from the Imperial Health Charity and the NIHR Imperial Biomedical Research Centre.

## Conflicts of Interest

The authors declare no conflicts of interest.

## Data Availability

The data that support the findings of this study are available on reasonable request from the authors.
